# Association between dementia and systemic rheumatic disease: A nationwide population-based study

**DOI:** 10.1371/journal.pone.0248395

**Published:** 2021-03-12

**Authors:** Hyemi Park, Dong-hyuk Yim, Bolormaa Ochirpurev, Sang-Yong Eom, In Ah Choi, Gawon Ju, Ji Hyoun Kim

**Affiliations:** 1 Department of Psychiatry, Chungbuk National University Hospital, Cheongju, Korea; 2 Center for Environmental Medicine, Chungbuk National University, Cheongju, Korea; 3 Department of Preventive Medicine, College of Medicine, Chungbuk National University, Cheongju, Korea; 4 Division of Rheumatology, Department of Internal Medicine, Chungbuk National University Hospital, Cheongju, Korea; 5 Division of Rheumatology, Department of Internal Medicine, Chungbuk National University College of Medicine, Cheongju, Korea; Kaohsuing Medical University Hospital, TAIWAN

## Abstract

**Objectives:**

Systemic rheumatic disease is characterized by autoimmunity and systemic inflammation and affects multiple organs. Few studies have investigated whether autoimmune diseases increase the risk of dementia. Herein, we evaluate the relationship between systemic rheumatic disease and dementia through a population-based study using the Korean National Health Insurance Service (NHIS) claims database.

**Methods:**

We conducted a nationwide population-based study using the Korean NHIS database, consisting of individuals who submitted medical claims from 2002–2013. Dementia was defined as having an acetylcholinesterase inhibitors (AChEIs) prescription along with symptoms satisfying the Alzhemier’s disease (AD) International Classification of Diseases (ICD)-10 codes (F00 or G30), or vascular dementia (VaD; ICD-10 or F01) criteria. Control subjects were matched to the dementia patients by age and sex. The study group was limited to those diagnosed with rheumatic disease at least 6 months prior to diagnosis of dementia. Rheumatic disease was defined by the following ICD-10 codes: Rheumatoid arthritis (RA: M05), Sjögren’s syndrome (SS: M35), systemic lupus erythematosus (SLE: M32), and Behcet’s disease (BD: M35.2).

**Results:**

Of the 6,028 dementia patients, 261 (4.3%) had RA, 108 (1.6%) had SS, 12 (0.2%) had SLE, and 6 (0.1%) had BD. SLE history was significantly higher in dementia patients (0.2%) than in controls (0.1%) and was associated with dementia (odds ratio [OR], 2.48; 95% confidence interval [CI], 1.19–5.15). In subgroup analysis, SLE significantly increased dementia risk, regardless of dementia type (AD: OR, 2.29; 95% CI, 1.06–4.91; VaD: OR, 4.54; 95% CI, 1.36–15.14). However, these associations were not sustained in the mild CCI or elderly group.

**Conclusion:**

SLE was independently associated with a higher risk of dementia, including AD and VaD when compared to the control group, even after adjustment. SLE patients (<65 years old) are a high-risk group for early vascular dementia and require screening for early detection and active prevention.

## Introduction

Dementia is a common mental illness associated with aging, and its increasing prevalence has led to an increased economic burden worldwide [1]. The elderly population of Korea is known to have the fastest increase in dementia prevalence in the world, with dementia being one of the most serious mental illnesses in the Korean population [2, 3]. According to the 2019 Korean dementia observatory, the estimated dementia population in 2018 was 748,946, with a predicted prevalence of 10.15%, and it is expected to exceed 2 million by 2039, with a prevalence of 12.3% [4].

Alzheimer’s disease (AD) is the most common cause of dementia in the elderly. Previous studies have reported that the accumulation of abnormal proteins, such as senile plaques, neurofibrillary tangles, and amyloid angiopathy, triggers neuroinflammation that activates innate immune responses. This, in turn, affects amyloid beta (Aβ) pathology and contributes to disease progression [5–7]. Vascular dementia (VaD) is the second most common cause of dementia, accompanied by varying degrees of ischemia or hemorrhagic cerebrovascular disease (CVD) [8]. VaD is known to be associated with atherosclerosis and arteriolosclerosis, which cause brain parenchymal lesions or chronic systemic inflammation [9]. There have been various studies for the risk factors of dementia, and Weber, et al [10] recently reported that the risk of dementia is increased in patients with osteoarthritis through meta-analysis.

Systemic rheumatic diseases such as rheumatoid arthritis (RA), Sjögren’s syndrome (SS), systemic lupus erythematosus (SLE), and Behcet’s disease (BD) are all caused by systemic inflammation and dysregulation of the immune system. The pathogenesis may be characterized by the presence of circulating autoantibodies and increasing levels of inflammatory cytokines or chemokines [11–13]. Previous studies have suggested that this inflammatory mechanism may play an important role in increasing the risk of cognitive function, leading to VaD and autoimmune related dementia [14–16]. However, few studies have investigated the association between dementia and systemic rheumatic diseases, and these few existing studies have reported contradictory results [17, 18]. Further work is, therefore, required to establish whether there are any associations between dementia and systemic rheumatic diseases.

This study aimed to investigate the risk of dementia in patients with systemic rheumatic diseases using the Korean National Health Insurance Service (NHIS)-National Sample Cohort (NSC) database.

## Materials and methods

### Database

We conducted a nationwide population-based study using the NHIS-NSC database of the Korean population who submitted medical care claims between 2002 and 2013. The Korean NHIS-NSC program covers approximately 97% of people in Korea for any medical procedure. Our dataset consisted of 1 million individuals who were selected by systematic stratified random sampling. The claim database included extensive information about diagnosis, demographics, health insurance type, death records, medical service and cost from all clinics and hospitals.

### Definitions of dementia patients and rheumatic disease

Dementia patients were defined as those prescribed with acetylcholinesterase inhibitors (AChEIs) at least once along with the symptoms outlined in the International Classification of Diseases (ICD)-10 code for dementia; AD (F00, G30), VaD (F01), and unspecified dementia (F03) were assigned accordingly. Those with dementia due to another causative disease (F02) were excluded from this study. To date, AChEIs is the only drug developed for the treatment of early dementia [19]. In this study, the history of AChEIs prescription was additionally confirmed to prevent errors caused by incorrect diagnosis code input and to increase the specificity when using national claims data. In addition, the index date for cases was defined as the date at which AChEIs were first prescribed. Dementia patients were matched at a 1:5 ratio with control group subjects who had never been treated for dementia. The control group was selected from the original population (n = 1,025,340) and matched according to age and sex. The presence or absence of rheumatic disease was confirmed in the dementia and control groups, respectively, and those diagnosed with rheumatic disease within 6 months of dementia were excluded from the analysis to allow for a wash out period.

Rheumatic diseases were identified individually using ICD-10 criteria. RA only included seropositive RA (M05), defined as a positive test for rheumatoid factor or anti-cyclic citrullinated peptide (CCP). SS (M35), SLE (M32), and BD (M35.2) were also defined using ICD-10 codes. The Korean government has provided financial support to patients with rare, incurable rheumatic diseases that are specially registered and have been managed since 2009. To be eligible for registration in the rare intractable diseases (RID) system, patients should meet the diagnostic criteria of each RID and are carefully reviewed by the corresponding healthcare institution. Therefore, diagnosis of patients with rheumatic disease registered in the RID system can be considered exceptionally reliable. These four diseases (RA, SS, SLE and BD) are also included in the Korean RID system. We collected information on the baseline demographics of patients and control subjects, including age, sex, region of residence, income level, and comorbidities (hypertension, diabetes, dyslipidemia, depression, cardiovascular disease, cerebrovascular disease, pulmonary disease, gastrointestinal disease, renal disease, connective tissue disorders, malignancy, and HIV).

### Selection of the study cohort

**Fig 1:**
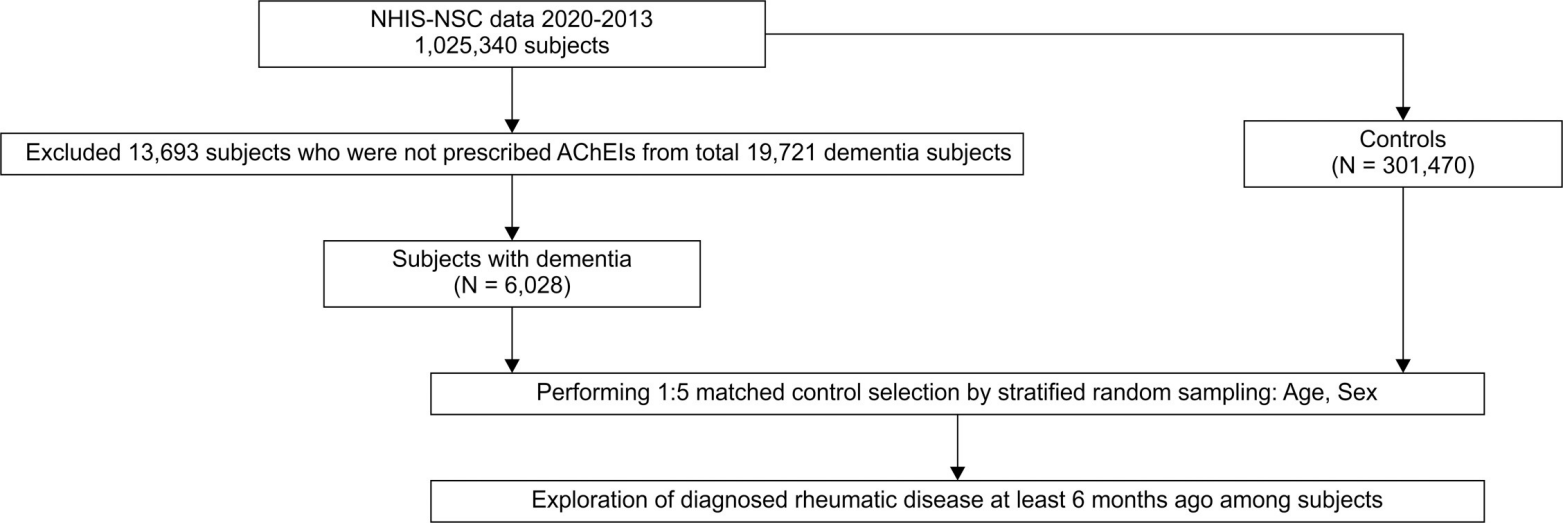
Flowchart for the selection of study participants from the National Health Insurance System of Korea database.

This study was a retrospective nested case-control study. Among patients diagnosed with dementia, 13,693 patients who were not prescribed AChEIs were excluded. We finally extracted 6,028 newly diagnosed dementia patients, including 5,045 (83.6%) with AD and 734 (12.2%) with VaD. Subjects younger than 20 years old or who could not be matched 1:5 with the control subjects by age and sex were also excluded. A total of 30,140 age-sex matched subjects were included in the control group. The flow chart for the selection of the study population is shown in Fig 1.

### Statistical analysis

The Chi-square test was used to compare the baseline characteristics of the dementia and control groups. Conditional logistic regression analyses were performed to test the association between rheumatic disease and dementia. Multivariate logistic analyses were used to adjust for confounding factors, such as income and comorbidities. The results generated from crude model (model 1) analysis, which were matched only by age and sex, and adjusted model (model 2) analysis, which controlled all risk factors that could affect dementia, such as income, residence, and comorbidities, as well as age and sex are presented. For the subgroup analysis, the subjects were divided into dementia type (AD or VaD), age group (<65 or ≥65 years old), and Charlson Comorbidity Index (CCI) [20] group according to disease severity. The CCI severity was classified as mild (CCI score, <3) or moderate to severe (CCI scores, ≥3). Statistical analysis was performed using SPSS Statistics Version 25.0 (IBM Corp., Armonk, NY, USA). A *P*-value less than 0.05 was considered statistically significant.

### Ethical statement

This study was exempted from ethical review according to the institutional review board of Chungbuk National University Hospital (CBNUH IRB No. 2019-03-018) as it is based on secondary data released for research purposes. Patient consent was not required for the present analysis.

## Results

In total, 6,028 newly diagnosed dementia subjects, including 5,045(83.6%) with AD and 734 (12.2%) with VaD, along with 30,140 age-sex matched control subjects, were included in the analysis. The demographic data and medical comorbidities of patients with and without dementia are presented in Table 1. In the dementia group, the female sex accounted for 4,221 (70.0%) of the patients. When subdivided by age, the results revealed that 4 (0.1%) of these patients were 20–39 years old, 459 (7.6%) were 40–64 years old, and 5,565 (92.3%) were ≥65 years old. After the matching process, we founded no significant differences in age or sex between the dementia group and control group.

**Table 1:** Baseline characteristic of dementia patients and age-sex matched control group.

	Dementia (N = 6,028)	Controls (N = 30,140)	*P*-value
Female sex, n (%)	4,221 (70.0)	21,105 (70.0)	1.000
Age group, n (%)			
20–39 years	4 (0.1)	20 (0.1)	1.000
40–64 years	459 (7.6)	2,295 (7.6)	
≥65 years	5,565 (92.3)	27,825 (92.3)	
AD, n (%)	5,045 (83.6)	-	
VaD, n (%)	734 (12.2)	-	
Residence city size, n (%)			
Seoul, metropolitan	1,053 (17.5)	5,319 (17.7)	0.459
Large cities*	1,420 (23.5)	6,877 (22.8)	
Other areas	3,555 (59.0)	17,944 (59.5)	
Household income**, n (%)			
High (>20%)	4,613 (76.5)	22,359 (74.2)	<0.001
Low (≤20%)	1,415 (23.5)	7,781 (25.8)	
Comorbidity, n (%)			
Hypertension	410 (67.1)	27,772 (61.2)	0.003
Diabetes	178 (29.1)	9,780 (26.7)	0.171
Dyslipidemia	98 (16.0)	4,744 (13.3)	0.052
Mood disorder	905 (8.0)	2,420 (15.0)	<0.001
Charlson Comorbidity Index, n (%)			
0	50 (0.8)	3,723 (12.4)	<0.001
1	559 (9.3)	6,635 (22.0)	
2	1,170 (19.4)	6,657 (22.1)	
≥3	4,249 (70.5)	13,125 (43.6)	

AD, Alzheimer’s disease; VaD, vascular dementia

*Large City: Busan, Daegu, Gwangju, Incheon, Daejeon, Ulsan

**Household income (divided into five quintiles and described in terms of the highest and lowest 20%).

However, there were significant differences in comorbidities. The distribution of hypertension (*P* = 0.003), SLE (*P* = 0.0136), and moderate to severe CCI (*P*<0.001) were increased, and depression (*P*<0.001) was decreased in the dementia group compared to the control group. In addition, the percentage of high-level income was also increased in the dementia group in comparison with the control group (*P*<0.001). There was no significant difference in geographic region between the two groups.

Table 2 shows the diagnostic history of systemic rheumatic disease in the dementia group compared to the control group. The results revealed that 261 (4.3%) of the dementia patients had RA, 108 (1.6%) had SS, 12 (0.2%) had SLE, and 6 (0.1%) had BD. In comparison, in the control group, 1,267 (4.2%) had RA, 481 (1.8%) had SS, 26 (0.1%) had SLE, and 51 (0.2%) had BD. History of SLE was significantly more common in dementia patients (0.2%) than in control subjects (0.1%), and was associated with dementia in both model 1 (odds ratio [OR], 2.31; 95% confidence interval [CI], 1.17–4.59, *P* = 0.016) and model 2 (OR, 2.48; 95% CI, 1.19–5.15, *P* = 0.0149) analyses. However, there were no significant differences between the dementia group and control group regarding the prevalence of RA (OR, 1.03; 95% CI, 0.90–1.18, *P* = 0.6353 in model 1; OR, 0.96; 95% CI, 0.83–1.11, *P* = 0.5481 in model 2), SS (OR, 1.13; 95% CI, 0.91–1.39, *P* = 0.2731 in model 1; OR, 1.1; 95% CI, 0.88–1.38, *P* = 0.3994 in model 2) or BD (OR, 0.59; 95% CI, 0.23–1.37, *P* = 0.2206in model 1; OR, 0.54; 95% CI, 0.22–1.29, *P* = 0.1641 in model 2).

**Table 2:** Odds ratios (ORs) for dementia according to prevalence of systemic rheumatic diseases.

	Overall dementia (N = 6,028)	Controls (N = 30,140)	Crude OR (95% CI, Model I)	*P*-value	Adjusted OR (95% CI, Model II)	*P*-value
Rheumatoid arthritis, n (%)	261 (4.3)	1,267 (4.2)	1.03 (0.90–1.18)	0.6353	0.96 (0.83–1.11)	0.5481
Sjögren’s syndrome, n (%)	108 (1.6)	481 (1.8)	1.13 (0.91–1.39)	0.2731	1.1 (0.88–1.38)	0.3994
Systemic lupus erythematosus, n (%)	12 (0.2)	26 (0.1)	2.31 (1.17–4.59)	**0.0164**	2.48 (1.19–5.15)	**0.0149**
Behcet’s disease, n (%)	6 (0.1)	51 (0.2)	0.59 (0.25–1.37)	0.2206	0.54 (0.22–1.29)	0.1641

Model 1 (crude): adjusted for age, sex; model 2: adjusted for age, sex, income, residence city size, comorbidities

Values in bold indicate a *P*-value <0.05

The relationship between rheumatic disease and dementia subtype (VaD, AD) was also investigated. In addition, since age and CCI, which are important risk factors in dementia, affect the association between rheumatic disease and dementia, the patients were grouped based on age and CCI to investigate the OR of dementia. Significant differences were only found in the SLE group (Table 3). Depending on the type of dementia, both AD (adjusted OR, 2.29; 95% CI, 1.06–4.91, *P* = 0.0341) and VaD (adjusted OR, 4.54; 95% CI, 1.36–15.14, *P* = 0.0137) showed elevated OR values. In the subgroup analysis, based on the CCI in the under 65-year old group, a significant association between dementia and a moderate to severe CCI was observed (OR, 6.79; 95% CI, 1.07–43.01 for overall dementia, OR, 3.75; 95% CI, 1.09–12.84 for VaD).

**Table 3:** Odds ratios (ORs) for dementia according to systemic lupus erythematosus by dementia type, CCI and age group.

Subgroup	OR (95% CI)					
	Adjusted OR	P-value	CCI<3	P-value	CCI ≥ 3	P-value
Overall dementia	2.48 (1.19–5.15)	**0.0149**	1.59 (0.19–13.12)	0.6683	2.9 (0.9–4.73)	0.0866
AD	2.29 (1.06–4.91)	**0.0341**	1.92 (0.23–15.93)	0.5462	1.62 (0.73–3.6)	0.2337
VaD	4.54 (1.36–15.14)	**0.0137**	-		3.75 (1.09–12.84)	**0.0356**
Age < 65yrs						
Overall dementia	5.67 (1.25–25.71)	**0.0244**			6.79 (1.07–43.01)	**0.0420**
AD	4.86 (0.98–24.04)	0.0528			2.47 (0.43–13.75)	0.3024
VaD	14.97 (2.46–91.06)	**0.0033**			7.76 (1.28–47.21)	**0.0261**
Age ≥65yrs						
Overall dementia	1.82 (0.77–4.27)	0.1701	2.26 (0.26–19.96)	0.4635	1.36 (0.56–3.31)	0.5031
AD	1.8 (0.74–4.38)	0.1931	2.73 (0.31–24.23)	0.3678	1.27 (0.47–3.41)	0.6399
VaD	1.88 (0.25–14.06)	0.5368	-		1.61 (0.22–12.1)	0.6436

Adjusted OR = adjusted for age, sex, income, residence city size, comorbidities

AD, Alzheimer’s disease; VaD, vascular dementia; CCI, Charlson Comorbidity Index

Values in bold indicate a *P*-value <0.05

When comparing the dementia group to the control group, no significant differences were observed in the adjusted ORs of RA, SS, and BD within each age-based subgroup, and each CCI related subgroup. The results are summarized in S1 Table (RA), S2 Table (SS) and S3 Table (BD). Although the statistical significance was not proved, the OR of BD was decreased in AD (0.55 in model 1; 95% CI 0.21–1.39, *P* = 0.2045) and increased in VaD (1.78 in model 1; 95% CI 0.43–7.45, *P* = 0.4305). These results were also consistent with the results generated by the age-based subgroup and CCI related subgroup analysis (S3 Table).

## Discussion

The purpose of this study was to determine the associations between systemic rheumatic diseases and the development of dementia. When compared to the control group, all dementia patients, including those with AD and VaD, were observed to have a high rate of systemic rheumatic disease. However, following subgroup analysis, only SLE was found to be associated with dementia, with no association being confirmed for RA, SS, or BD.

In this study, SLE was associated with a 2.4-fold greater likelihood of having a dementia diagnosis, even after excluding the effects of other comorbidities. SLE is a disease that has been linked with dementia in the past, and in 1999, the American College of Rheumatology (ACR) termed the disease, “neuropsychiatric SLE” (NPSLE), and proposed 19 symptoms including stroke, depression, psychosis, and cognitive impairment [21]. The possible mechanisms behind SLE have been discussed and include the passage of brain reactive autoantibodies and inflammatory cells through the blood brain barrier (BBB), thereby causing damage to the brain, as well as diffuse vasculopathy, and atherosclerosis [22, 23].

The mechanisms behind chronic inflammation, including microglia activation by aggregated Aβ, and activation of the complement system involved in the pathogenesis of AD, have been described [24]. Cell destruction by phagocytosis, membrane attack complex (MAC) [25] by opsonizing component markers, as well as the proinflammatory cytokines (IL-1, IL6, TNF-α, and interferon-γ) [26] produced by these innate immune responses affect the disease progression and severity of dementia.

VaD, which is not a specific diagnosis but rather a syndrome encompassing diseases caused by damage to the blood supply to the brain [27], is the second most common cause of dementia. VaD is traditionally thought to be associated with cardiovascular risk factors, but immunological damage to brain blood vessels and neuronal cells is also involved [27]. In addition, previous neuropathological studies have suggested that characteristic inflammation of the neurovasculature seen in AD is also an important factor in its pathogenesis. Accumulation of Aβ deposits reduces cerebral blood flow and causes chronic hypoxia and nerve damage [6, 28]. Transport and accumulation of Aβ in the brain are accelerated through the blood brain barrier [29]. These results indicated that the pathogeneses of AD and VaD are not completely independent, and further studies on the mechanism behind the increased risk of dementia with SLE during this complex process should be performed. It is well known that antiphospholipid syndrome (APS) is also commonly associated with SLE and is associated with repetitive thromboembolic events, including stroke, and results in VaD [30]. A non-ischemic mechanism has also been suggested through animal experiments; the destruction of the BBB is accelerated, thereby increasing its permeability and the resulting inflammatory cells produce cytokines [31].

Another possible mechanism is via anti-N-methyl-D-aspartate (NMDA) antibodies. NMDA receptors are expressed in the hippocampus, amygdala, and hypothalamus of the brain, and anti-NMDA antibodies are known to be found in 14–35% of SLE patients. It has been demonstrated, through animal models, that these antibodies activate NMDA receptors, leading to a state of ‘excitotoxicity’, ultimately reduce NMDA receptor expression in hippocampal neurons, and contribute to a cognitive decline [32]. It is also possible that long-term or high-dose glucocorticoid treatment to reduce inflammation in SLE reduces the hippocampus volume through the hypothalamic–pituitary–adrenal axis, thereby contributing to the cognitive decline [33].

The results of this study suggest that the risk of VaD was 15 times higher in SLE patients that were aged 65 years or younger. Previous studies have shown that the average age of dementia in SLE patients with APS is approximately 50 years, which is consistent with the results of this study, that indicated the risk of VaD is increased in SLE patients under 65 years of age [34]. In this case, autoantibodies (anti-cardiolipin, anti-β2-glycoprotein-1, and a lupus anticoagulant) are often accompanied by cardiovascular disease risk factors; however, this unfortunately could not be identified using the medical claim data.

RA is a chronic autoimmune disease that mainly causes joint pain and inflammation. Extra-articular manifestation occurs in approximately 40% of patients, but central nervous system involvement is relatively rare [35]. It is known that cerebral vasculitis, which can increase the risk of VaD, is an uncommon and serious complication of seropositive RA, resulting from pro-inflammatory mediators (cytokines, chemokines, and adhesion molecules) and autoantibodies against endothelial cell components [36]. Several previous studies have shown an increase in dementia in RA patients [17, 37–39], while other studies have shown a decrease in dementia risk in RA patients [40]. The present study did not show that dementia risk was increased in RA patients.

Unlike our findings, previous studies [41, 42] reported an increased risk of AD in primary SS. The adjusted hazard ratio was 2.69 in primary SS patients compared to the control [41, 42]. The presence of anti-Ro (SS-A) antibodies was mainly associated with systemic clinical manifestation. Additionally, an anti-muscarinic acetylcholine receptor (mAChR) autoantibody was recently discovered in primary SS patients, and M1 mAChR regulates Aβ processing and reduces protein hyperphosphorylation and is therefore an important therapeutic target for AD therapy that potentially restores cognitive function. Previous studies [41, 42] analyzed using only the ICD code. However, in order to increase the reliability of the diagnosis of dementia, our study analyzed patients who took acetylcholinesterase inhibitors, a representative dementia medicine when defining dementia. Therefore, compared to previous studies, we were more likely to include patients with a moderate or higher degree of dementia that required medication rather than patients with a mild cognitive impairment.

It has been reported that neuro-behcet’s disease (NBD), which is characterized by an elevated β-cell activating factor level [43], indicates chronic cognitive impairment. Meta-analysis was performed in connection with various phenotypes of NBD, and dementia risk was found to be increased [44]. Dementia in BD patients was mainly associated with vasculitis or vasculopathy, and the relationship with AD is still unknown. Our study also did not show significant results on the association between dementia and BD.

The limitations of this study should be noted. Firstly, the dataset did not provide demographic information for risk factors of dementia such as education level or family history. The second limitation was that laboratory information, such as Apolipoprotein E genotyping, inflammatory biomarkers, auto antibodies of rheumatic diseases, and evaluation of disease activity, were not included. Third, this study relied on the Korean medical insurance database which have various forms depending on the type of employment, family support and type of subscription, there is therefore a possibility that the results do not reflect the exact economic status. Nevertheless, an increase in the dementia rate in the high-income group was likely to be associated with an increase in hospital utilization (mean 479.7±407.1 days in dementia group vs 382.8±362.1 days in the control group, p <0.0001) and the trend was consistent with the results of previous studies [45, 46]. Finally, drug-related effects, including nonsteroidal anti-inflammatory drugs, steroids, conventional disease-modifying anti-rheumatic drugs (DMARDs), biologic DMARDs, and targeted synthetic DMARDs were not evaluated. However, this study was a population-based study, and is considered to be the first study to investigate the overall risk of representative systemic rheumatic diseases in the development of dementia in patients in Korea.

In conclusion, in this case-controlled study, we investigated the associations between systemic rheumatic diseases and dementia. In particular, the results revealed a significant association between dementia and SLE compared to controls. SLE patients (younger than 65) were at a high-risk group for early VaD and require screening for early detection and active prevention. We believe that providing treatment to control disease activity, along with risk factor management, may help prevent dementia in SLE patients.

## Supporting information

S1 Table Odds ratios (ORs) for dementia according to rheumatoid arthritis stratified by dementia type, CCI and age group. (DOCX)

S2 Table Odds ratios (ORs) for dementia according to Sjögren’s syndrome stratified by dementia type, CCI and age group. (DOCX)

S3 Table Odds ratios (ORs) for dementia according to Behcet’s disease stratified by dementia type, CCI and age group. (DOCX)
