# Supplementary material for: Association between dementia and systemic rheumatic disease: A nationwide population-based study
Source: PLoS One. 2021 Mar 12;16(3):e0248395. doi: 10.1371/journal.pone.0248395 (PMC7954284; doi:10.1371/journal.pone.0248395)
Supplement: S1 Table — (DOCX) [file pone.0248395.s001.docx]

**S1 Table.** Odds ratios (ORs) for dementia according to Rheumatoid arthritis stratified by dementia type, CCI and age group

| Type (Rheumatoid arthritis) | | OR (95% CI) | | | | | | |
| --- | --- | --- | --- | --- | --- | --- | --- | --- |
|  |  | Adjusted OR | | P-value | CCI < 3 | *P*-value | CCI ≥ 3 | *P*-value |
| Overall dementia | | 0.96 (0.83-1.11) | | 0.5481 | 1.01 (0.73-1.46) | 0.8626 | 0.85 (0.72-1.01) | 0.0582 |
| AD | | 0.95 (0.81-1.11) | | 0.5065 | 1.06 (0.73-1.55) | 0.7458 | 0.89 (0.75-1.05) | 0.1745 |
| VaD | | 1.18 (0.83-1.68) | | 0.3496 | 1.44 (0.52-3.95) | 0.48 | 1.06 (0.73-1.55) | 0.7484 |
| Age ≥65yrs | |  | |  |  |  |  |  |
| Overall dementia | | 0.98 (0.84-1.14) | | 0.7675 | 1.08 (0.76-1.53) | 0.6672 | 0.92 (0.79-1.09) | 0.3395 |
| AD | | 0.96 (0.82-1.12) | | 0.5905 | 1.11 (0.76-1.61) | 0.6034 | 0.84 (0.71-1.01) | 0.0635 |
| VaD | 1.21 (0.84-1.74) | | 0.2965 | | 1.46 (0.54-4.09) | 0.4409 | 1.12 (0.76-1.64) | 0.5745 |

Adjusted OR= adjusted for age, sex, income, residence city size, comorbidities, AD=Alzheimer’s disease; VaD=vascular dementia; CCI=Charlson Comorbidity Index
