# Supplementary material for: Association between dementia and systemic rheumatic disease: A nationwide population-based study
Source: PLoS One. 2021 Mar 12;16(3):e0248395. doi: 10.1371/journal.pone.0248395 (PMC7954284; doi:10.1371/journal.pone.0248395)
Supplement: S2 Table — (DOCX) [file pone.0248395.s002.docx]

**S2 Table.** Odds ratios (ORs) for dementia according to Sjögren’s syndrome stratified by dementia type, CCI and age group

| Type (Sjögren’s syndrome) | | OR (95% CI) | | | | | | |
| --- | --- | --- | --- | --- | --- | --- | --- | --- |
|  |  | Adjusted OR | *P*-value | CCI < 3 | *P*-value | CCI ≥ 3 | *P*-value |  |
| Overall dementia | | 1.1(0.88-1.38) | 0.3994 | 1.14 (0.61-2.15) | 0.6788 | 1.07 (0.84-1.38) | 0.5833 |  |
| AD | | 1.11(0.87-1.41) | 0.3901 | 1.01 (0.48-2.09) | 0.9903 | 1.03 (0.8-1.32) | 0.8470 |  |
| VaD | | 1.15(0.65-2.05) | 0.6310 | 1.5 (0.2-10.98) | 0.6916 | 1.08 (0.59-1.97) | 0.7953 |  |
| Age ≥65yrs | |  |  |  |  |  |  |  |
| Overall dementia | | 1.12(0.88-1.41) | 0.3566 | 1.09 (0.56-2.1) | 0.8104 | 1.02 (0.8-1.3) | 0.8880 |  |
| AD | | 1.12(0.87-1.43) | 0.3837 | 1.51 (0.51-2.19) | 0.8939 | 1.11 (0.85-1.45) | 0.4553 |  |
| VaD | 1.17(0.64-2.12) | | 0.6168 | 1.54 (0.21-11.33) | 0.6718 | 1.03 (0.55-1.92) | 0.9265 |  |

Adjusted OR= adjusted for age, sex, income, residence city size, comorbidities, AD=Alzheimer’s disease; VaD=vascular dementia; CCI=Charlson Comorbidity Index
