# Supplementary material for: Association between dementia and systemic rheumatic disease: A nationwide population-based study
Source: PLoS One. 2021 Mar 12;16(3):e0248395. doi: 10.1371/journal.pone.0248395 (PMC7954284; doi:10.1371/journal.pone.0248395)
Supplement: S3 Table — (DOCX) [file pone.0248395.s003.docx]

**S3 Table**. Odds ratios (ORs) for dementia according to Behcet’s disease stratified by dementia type, CCI and age group

| Type (Behcet’s disease) | | OR (95% CI) | | |  |
| --- | --- | --- | --- | --- | --- |
|  |  | Adjusted OR | *P*-value | CCI ≥ 3 | *P*-value |
| Overall dementia | | 0.54 (0.22-1.29) | 0.1641 | 0.48 (0.2-1.15) | 0.0987 |
| AD | | 0.55 (0.21-1.39) | 0.2045 | 0.52 (0.2-1.32) | 0.1693 |
| VaD | | 1.78 (0.43-7.45) | 0.4305 | 1.57 (0.37-6.62) | 0.5419 |
| Age ≥65yrs | |  |  |  |  |
| Overall dementia | | 0.62 (0.26-1.49) | 0.2851 | 0.6 (0.25-1.43) | 0.2504 |
| AD | | 0.61(0.24-1.58) | 0.3118 | 0.54 (0.21-1.4) | 0.2027 |
| VaD | 2.15 (0.51-9.07) | | 0.2969 | 2.05 (0.49-8.63) | 0.3263 |

Adjusted OR= adjusted for age, sex, income, residence city size, comorbidities, AD=Alzheimer’s disease; VaD=vascular dementia; CCI=Charlson Comorbidity Index
